# Sepsis causes neutrophil infiltration in muscle leading to muscle atrophy and weakness in mice

**DOI:** 10.3389/fimmu.2022.950646

**Published:** 2022-10-31

**Authors:** Nobuto Nakanishi, Yuko Ono, Yusuke Miyazaki, Naoki Moriyama, Kazumichi Fujioka, Kimihiro Yamashita, Shigeaki Inoue, Joji Kotani

**Affiliations:** ^1^ Division of Disaster and Emergency Medicine, Department of Surgery Related, Kobe University Graduate School of Medicine, Kobe, Japan; ^2^ Department of Pediatrics, Kobe University Graduate School of Medicine, Kobe, Japan; ^3^ Division of Gastrointestinal Surgery, Department of Surgery, Kobe University Graduate School of Medicine, Kobe, Japan

**Keywords:** muscle atrophy, sepsis, neutrophil, fibrosis, mice

## Abstract

**Background:**

Sepsis-induced muscle atrophy leads to prolonged physical dysfunction. Although the interaction of muscle atrophy and macrophage has been reported in sepsis, the role of neutrophils in muscle atrophy has not been thoroughly investigated. This study sought to investigate the long-term changes in muscle-localized neutrophils after sepsis induction and their possible role in sepsis.

**Methods:**

Sepsis was induced in seven-week-old male C57BL/6J mice 8-12 (cecal slurry [CS] model) *via* intraperitoneal injection of 1 mg/g cecal slurry. The percentage change in body weight and grip strength was evaluated. The tibialis anterior muscles were dissected for microscopic examination of the cross-sectional area of myofibers or Fluorescence-activated cell sorting (FACS) analysis of immune cells. These changes were evaluated in the following conditions: (1) Longitudinal change until day 61, (2) CS concentration-dependent change on day 14 at the low (0.3 mg/g), middle (1.0 mg/g), and high (2.0 mg/g) concentrations, and (3) CS mice on day 14 treated with an anti-Ly6G antibody that depletes neutrophils.

**Results:**

Body weight and grip strength were significantly lower in the CS model until day 61 (body weight: 123.1% ± 1.8% vs. 130.3% ± 2.5%, p = 0.04; grip strength: 104.5% ± 3.8% vs. 119.3% ± 5.3%, p = 0.04). Likewise, cross-sectional muscle area gradually decreased until day 61 from the CS induction (895.6 [606.0–1304.9] μm^2^ vs. 718.8 [536.2–937.0] μm^2^, p < 0.01). The number of muscle-localized neutrophils increased from 2.3 ± 0.6 cell/mg on day 0 to 22.2 ± 13.0 cell/mg on day 14, and decreased thereafter. In terms of CS concentration–dependent change, cross-sectional area was smaller (484.4 ± 221.2 vs. 825.8 ± 436.2 μm^2^ [p < 0.001]) and grip strength was lower (71.4% ± 12.8% vs. 116.3% ± 7.4%, p = 0.01) in the CS High group compared with the control, with increased neutrophils (p = 0.03). Ly6G-depleted mice demonstrated significant increase of muscle cross-sectional area and grip strength compared with control mice (p < 0.01).

**Conclusions:**

Sepsis causes infiltration of neutrophils in muscles, leading to muscle atrophy and weakness. Depletion of neutrophils in muscle reverses sepsis-induced muscle atrophy and weakness. These results suggest that neutrophils may play a critical role in sepsis-induced muscle atrophy and weakness.

## Introduction

Due to advancements in medical treatments, sepsis mortality has decreased by 53% from 1990 to 2017 (1). However, approximately one-third of sepsis survivors have persistent physical impairment 6 months after hospital discharge (2). The loss of skeletal muscle mass during the acute phase is one of the causes of prolonged physical impairment. Sepsis causes skeletal muscle mass loss of 10%–20% in a week, which is associated with functional decline and mortality (3). Although muscle atrophy has been reported in numerous studies, its underlying mechanism has not been elucidated.

One poorly understood field is the interaction between muscle atrophy and the immune system (4). Immune effector cells are involved in muscle tissue destruction and construction (5). While most studies have focused on the role of macrophages in muscle atrophy, neutrophils also play important roles in muscle atrophy (6). Neutrophils are frontline cells that combat microbes by releasing antimicrobial agents and are essential for maintaining tissue homeostasis (7). In exercise- (8) or toxin-induced muscle injuries (9), neutrophil levels were increased in human muscle tissue observed under a microscope. Neutrophil activation can lead to muscle atrophy because neutrophil depletion contributes to reduced skeletal muscle damage and atrophy in a non-sepsis model (10).

However, in sepsis, the interaction between muscles and the immune system is poorly understood. Few previous studies, in our opinion, have investigated the immunologic mechanism in sepsis-induced muscle atrophy or injury. We hypothesized that muscle-localized neutrophils play an important role in sepsis-induced skeletal muscle atrophy. The purpose of the study is to address the longitudinal changes of muscle-localized neutrophils after sepsis and elucidate the interaction between neutrophils and muscle atrophy and weakness.

## Material and methods

### Ethics

This animal experiment was approved in July 2021 by the Committee on the Ethics of Animal Experiments of Kobe University Graduate School of Medicine (P210704). All experimental procedures were carried out in accordance with the recommendations of the International Expert Consensus Initiative for Improvement of Animal Modeling in Sepsis (11).

### Animals

Seven-week-old male C57BL/6J mice were purchased from Charles River (St-Constant, Quebec, Canada). The mice were housed in cages, in groups of five, with a 12-h light-dark cycle and a temperature of 22°C, with water and food ad libitum. Before beginning the test, the mice were acclimatized for a week.

### Preparation of cecal slurry

As previously reported, cecal slurry (CS) was prepared (12). To induce polymicrobial sepsis, male Institute for Cancer Research mice aged 8 to 12 weeks were intraperitoneally injected with CS (13). After the mice were sacrificed, the cecum was harvested and ground through a 70 μm mesh cell strainer (EASYstrainer™, Greiner Bio-One, Kremsmünster, Austria). The sample was combined with 1–2 mL of sterile phosphate-buffered saline (PBS, Wako, Osaka, Japan) and filtered twice. Following this, the mixture was centrifuged at 11,000 rpm for 1 min. After removing the supernatant, the residue was mixed with 15% glycerol-PBS to achieve a concentration of 500 mg/mL. The sample (400–500 µL) was then transferred to cryogenic biobanking tubes (Greiner Bio-one, Kremsmünster, Austria) and stored at −80°C until use.

### Sepsis model by CS injection

**Figure 1:**
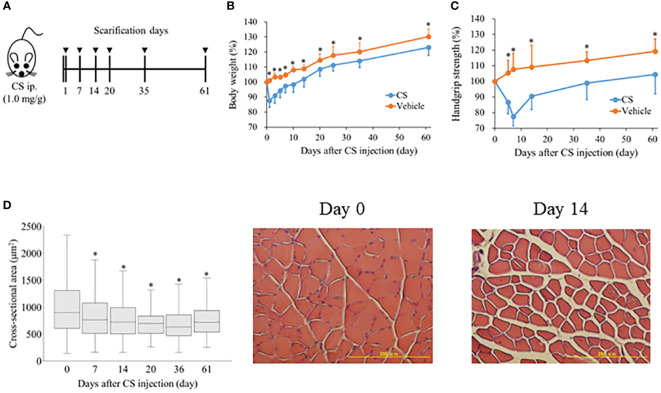
Sepsis decreases body weight, grip strength, and muscle atrophy in mice. **(A)** Shema showing study design. Mice were injected 1.0 mg/g of CS intraperitoneally and body weight, grip strength were evaluated from day 1 to 61 (n = 9 in control, n = 22 in CS). To measure cross-sectional area of the anterior tibialis, mice were sacrificed on days 7, 14, 20, 35, and 61 after CS injection. **(B)** Body weight was measured with the sample size (CS/control) at day 0 (22/9), 1 (22/9), 3 (19/8), 5 (19/8), 7 (19/8), 10 (16/7), 14 (16/7), 20 (13/6), 25 (13/6), 35 (13/6), and 61 (10/5). **(C)** Grip strength was measured with the sample size (CS/control) at 0 (20/8), 5 (20/8), 7 (20/8), 14 (17/8), 35 (11/6), and 61 (8/5). **(D)** Histological change in the cross-sectional area of anterior tibialis following sepsis induction. Cross-sectional area of the anterior tibialis decreased after sepsis induction. * Statistical significance at *p* < 0.05 versus control.

For the controls, intraperitoneal injection of 1 mg/g bodyweight CS or an equal volume of vehicle (15% glycerol-PBS) was administered to induce sepsis. Mice were anesthetized with isoflurane inhalation and sacrificed on the days specified after CS injection (**Figure 1A**). Flow cytometry and histology were performed on the right or left tibialis anterior muscle samples, respectively.

To investigate the CS concentration-dependent change, CS was intraperitoneally injected at three different concentrations: 0.3 mg/g (n = 3), 1.0 mg/g (n = 3), 2.0 mg/g bodyweight (n = 4), and vehicle (n = 3). On day 14 after CS induction, we compared the percentage and the number of neutrophil, monocyte, and macrophage in the right tibialis anterior muscle; grip strength; and the cross-sectional area of the left tibialis anterior muscle.

### Grip strength

A grip strength meter was used to measure grip strength (MK-380Si; Muromachi Kikai, Tokyo, Japan). As previously described, the grip strength test was carried out with minor modifications (14, 15). In brief, mice were allowed to use their front paws to grab a horizontal bar mounted on the gage, and their tails were pulled back. Grip strength was measured three times before and after CS injection, and the highest value was recorded.

### Muscle histology

Tibialis anterior muscles were carefully dissected from both legs of five CS and two control mice on days 7, 14, 20, 35, and 61 following sepsis induction. For at least one day, the tibialis anterior muscle in the left leg was immersed in 4% paraformaldehyde containing 0.2% picric acid. The tibialis anterior muscle was paraffin-embedded and sectioned at a thickness of 5 μm. The section was then stained with a hematoxylin and eosin (H & E) solution (Wako, Osaka, Japan). The specimen was examined with a light microscope equipped with a camera (Olympus BX43, Tokyo, Japan) (Olympus DP70, Tokyo, Japan). Next, ImageJ software was used to quantify the captured image at 20 times magnification (National Institutes of Health, Bethesda, MD, USA). To calculate the median myofiber cross-sectional area, approximately 300 myofibers were measured in at least five fields of view per group.

### Identification of immune cells in muscle

The right leg’s sectioned anterior tibialis muscle was minced with scissors. The minced muscle was combined with 10 mL of RPMI containing 1 mg/mL collagenase type 2 and 0.1 mg/mL Deoxyribonuclease 1. (Worthington, Lakewood, New Zealand). In a shaking incubator, the sample was shaken at 200 rpm for 30 min at 37°C (BR-13FP, Taitec Co, Saitama, Japan). Following the addition of PBS containing 0.1% bovine serum albumin (BSA), the digested tissue was filtered through a 70 μm mesh (EASYstrainer™, Greiner Bio-One, Kremsmünster, Austria). A Countess II FL cell counter was used to count the cells (Thermo Fisher, Waltham, MA, USA). The following antibodies were used in this study: anti-CD45, anti-CD11b, anti-F4/80, anti-GR1, anti-Ly6G, anti-Ly6C, anti-CCR2 anti-CXCR2 (**Table S1** in the supplemental file). The gating strategy is shown in **Figure S1**, and individual immune cell subtypes were identified by the following combinations. Neutrophil: CD45^+^, CD11b^+^, F4/80^−^, GR1^+^, Ly6G^+^, Ly6C^Int^; Monocyte: CD45^+^, CD11b^+^, F4/80^−^, GR1^+^, Ly6G^−^, Ly6C^+^; Macrophage: CD45^+^, CD11b^+^, F4/80^+^. Flow cytometry was performed using FACS Verse (BD Biosciences, San Jose, CA, USA), and the data were analyzed using Flow Jo software v10 (Tree Star Inc, Ashland, OR, USA).

### Blood sampling and measurements of immune cells

Blood samples were collected from three CS mice and one control mouse on days 0.5, 3, 8, 15, 21, and 30 following CS injection. Flow cytometry was used to count neutrophils, monocytes, and macrophages in blood. Blood was drawn from the inferior vena cava with a heparinized syringe and deposited into a tube using a 23G needle (Terumo, Tokyo, Japan). Blood samples were layered on Histopaque-1119 (Sigma Aldrich, St Louis, MO, USA) to isolate peripheral blood mononuclear cells after being diluted with 0.1% BSA in PBS (PBMCs). The samples were centrifuged at 2000 rpm for 20 minutes to collect PBMCs. Incubation was conducted in a hemolysis buffer at 37°C for 10 min (139.5 mM NH_4_Cl and 1.7 mM Tris-HCl). The PBMCs were washed in PBS containing 0.1% BSA, blocked with mouse Fc-blocker (Miltenyi Biotec, Bergisch Gladbach, Germany), and incubated with the Abs mixture for 20 minutes at 4°C. The same gating strategy was used with the muscle tissue. Flow cytometry and analysis were then carried out using FACS Verse and Flow Jo software v10, respectively.

### Neutrophil depletion

Five mice were intraperitoneally administered with either 200 μg of Ly6G-specific monoclonal antibody to deplete Ly6G+ cells or IgG2a isotype control antibodies (InVivoPlus anti-mouse Ly6G, Bioxcell, St Louis, NH, USA) 5, 8, 11, 13 days after CS induction. One mouse on each side was cheek bled one day after the first Ly6G-specific monoclonal antibody induction and on the day of scarifice at day 14 to confirm the depletion of Ly6G^+^ cells *via* flow cytometry. On day 14, after CS induction, we compared the percentage of neutrophils in right tibialis anterior muscle, grip strength, and the cross-sectional area of left tibialis anterior muscle.

### Immunohistochemistry

Immunohistochemistry was used to investigate neutrophil localization and the presence of NETs. The sections were deparaffinized with xylenes, rehydrated with graded ethanol, and briefly washed in distilled water. Then, for 30 min, it was blocked with PBS containing 10% BSA to prevent nonspecific immunoglobulin binding. The section was stained for 1 hour at room temperature with a rabbit anti-citrullinated-histone H3 antibody (1:1000, ab5103; Abcam, Cambridge, UK), followed by one-hour incubation with a second antibody (green, 1:500, goat anti-rabbit Alexa 488, Cell Signaling Technology, Danvers, MA, USA). Another antibody, anti-Ly-6G (Gr-1) (red, 1:200, RB6-8C5, Merck, Darmstadt, Germany), was used for 1 hour. Finally, the sections were stained for 30 minutes with 4, 6-diamidino-2-phenylindole (DAPI, blue, 1:500, Dojin, Kumamoto, Japan). Three representative images (20× magnification) were taken in a muscle section using the BZ-X100 microscope (Keyence, Tokyo, Japan). Colocalization of Gr-1, histone H3, and DAPI was considered for NETs.

### Statistical analysis

Continuous data were expressed as the mean ± standard deviation or medians (interquartile range), and compared using the t-test or Mann-Whitney U test, as appropriate. All statistical tests were two-tailed, and statistical significance was defined as a p-value <0.05. For statistical analysis, we used JMP statistical software version 13.1.0 (SAS Institute Inc., Cary, NC, USA).

## Results

### Sepsis decreases body weight, grip strength, and muscle cross-sectional area

After CS injection, we investigated these changes to confirm the longitudinal change in body weight, hand grip strength, and muscle mass. Following CS induction, body weight decreased considerably (CS model vs. control; 87.7% ± 4.4% vs. 101.1% ± 1.2%, p < 0.01) (**Figure 1B**), and remained significantly lower in the CS model until day 61 (CS model vs. control; 123.1% ± 1.8% vs. 130.3% ± 2.5%, p = 0.04). In contrast, hand grip strength in the CS model decreased until day 7 (CS model vs. control; 77.5% ± 1.8% vs. 107.7% ± 2.8%, p < 0.01) (**Figure 1C**), and gradually increased. Until day 61, the hand grip strength was statistically significantly lower in the CS model than control (CS model vs. control; 104.5% ± 3.8% vs. 119.3% ± 5.3%, p = 0.04). The cross-sectional area of the anterior tibialis was 895.6 (606.0–1304.9) μm^2^ at day 0 (**Figure 1D**). On day 7, the cross-sectional area was significantly reduced (762.5 [510.2–1076.1] μm^2^, p < 0.01) until day 61 (718.8 [536.2–937.0] μm^2^, p < 0.01). The typical histologic change in the muscle tissue is shown in **Figure 1D**. These results suggest that that muscle atrophy and weakness lasted one to two months after sepsis.

### Neutrophils infiltrate muscles during the subacute phase after sepsis

**Figure 2:**
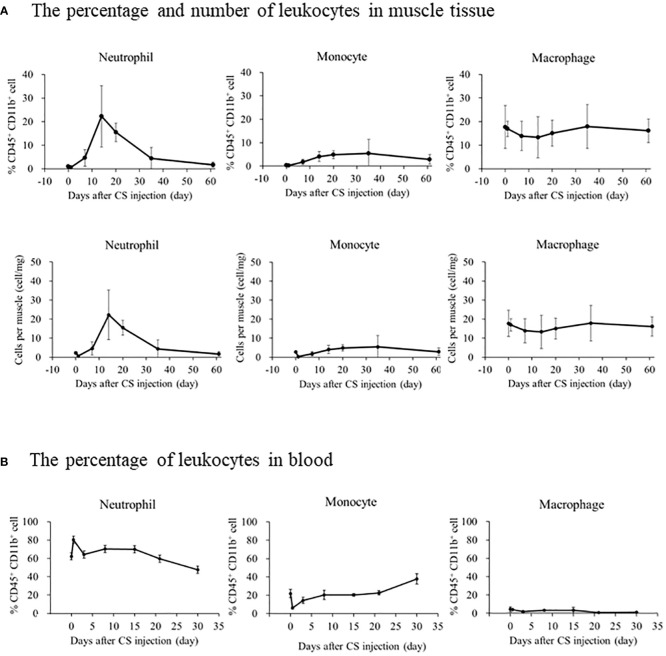
The longitudinal change in immune effector cells in muscle or blood after CS injection in mice. **(A)** The upper graph depicts the percentage change in immune effector cells in CD45^+^CD11b^+^ cells in the muscle, and the lower graph depicts the number of immune effector cells identified per muscle. Number of samples were Five CS and two control mice at each measurement day. **(B)** The percentage of immune effector cells in CD45^+^CD11b^+^ cells in the blood. Number of samples were three CS and one control mice at each measurement day.

We investigated immune effector cells in the muscle and blood to determine the longitudinal change in immune effector cells and the difference between muscles and blood. A typical depiction of flow cytometry is shown in **Figure S4** in which muscle-localized Gr-1^+^ cells were compared between naive and CS models 14 days after injection. Gr-1^+^ cells, as well as CD11b^+^ CD45^+^ cells, were detected in high numbers in the CS model. On day 14 of the CS model, immunohistochemistry detected several Gr-1^+^ cells (**Figure S5**). The percentage or number of cells displaying the trend of muscle-localized immune effector cells was shown in **Figure 2A**. The neutrophil ratio per CD45^+^ CD11b^+^ cells increased from 1.0% ± 0.5% on day 0 to 22.2% ± 13.0% on day 14 and 15.5% ± 3.9% on day 20. Likewise, the number of neutrophils per muscle increased from 2.3 ± 0.6 cell/mg on day 0 to 22.2 ± 13.0 cell/mg on day 14 and 15.5 ± 3.9 cell/mg on day 20. Contrary to the number of neutrophils, the number of monocytes and macrophages did not peak on day 14 (4.1 ± 2.1 cell/mg and 13.3 ± 8.8 cell/mg, respectively). Unlike the muscle-localized immune effector cells, the neutrophil ratio per CD45^+^ CD11b^+^ in the blood peaked at day 0.5 (80.3% ± 2.0%), and gradually decreased until day 30 (47.5% ± 8.9%) (**Figure 2B**). Until day 35, the number of monocytes slightly increased (37.8% ± 5.6%), and macrophages decreased (1.4% ± 0.1%). These findings suggest that neutrophils infiltrate the muscle during the subacute phase after sepsis, while neutrophils in blood increases during the acute phase after sepsis.

### Sepsis induces neutrophil infiltration, muscle atrophy, and weakness in a CS-dose dependent manner

**Figure 3:**
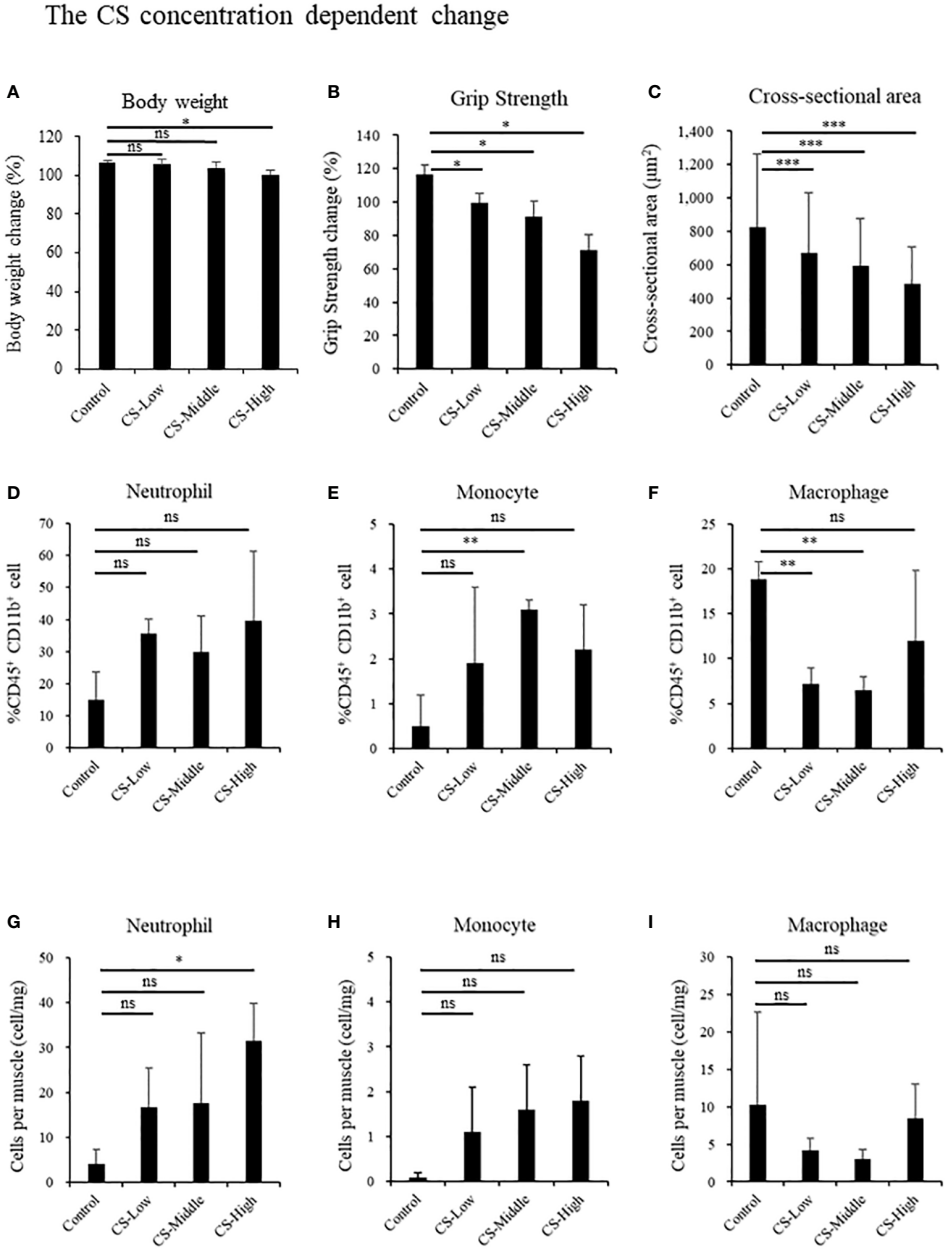
Sepsis induces neutrophil infiltration, muscle atrophy, and weakness by CS-dose dependent manner in mice. Body weight, grip strength, and the cross-sectional area of the tibialis anterior muscle, and immune effector cells at day 14 in the different concentrations of low: 0.3 mg/g (n = 3), middle: 1.0 mg/g (n = 3), high: 2.0 mg/g (n = 4). **(A)** Body weight, **(B)** Grip strength, **(C)** Cross-sectional area of the tibialis anterior muscle, percentages of **(D)** Neutrophils, **(E)** Monocyte, **(F)** Macrophage, population of **(G)** Neutrophils, **(H)** Monocyte, **(I)** Macrophage. Statistical significance versus control at *P < 0.05, **p < 0.01, ***p < 0.001, ns = not significant (p > 0.05).

We investigated the influence of CS concentration on body weight (**Figure 3A**), grip strength, muscle cross-sectional area, and muscle localized immune cells. Half of the mice injected with the CS dose of 2.0 mg/g died until day 14, and two mice remained for comparison. Grip strength dose-dependently decreased in the CS model (Control: 116.3% ± 7.4%; CS-Low: 99.3% ± 7.0% [p = 0.04]; CS-Middle: 91.1% ± 11.9% [p = 0.04]; and CS-High: 71.4% ± 12.8% [p = 0.01], **Figure 3B**). Cross-sectional area dose-dependently decreased in the CS model (Control: 825.8 ± 436.2 μm^2^; CS-Low: 668.5 ± 362.6 μm^2^ [p < 0.001]; CS-Middle: 591.4 ± 286.1 μm^2^ [p < 0.001]; and CS-High: 484.4 ± 221.2 μm^2^ [p < 0.001], **Figure 3C**). Contrary to monocyte and macrophage, the neutrophil ratio per CD45^+^ CD11b^+^ cells was the highest in CS-High group among concentration-dependent CS mice (CS-High vs. Control; 39.6% ± 21.7% vs. 14.9% ± 8.8%, p = 0.07, **Figures 3D–F**), and the number of neutrophils per muscle was the highest in CS-High (CS-High vs. Control; 31.5 ± 8.3 cell/mg vs. 4.1 ± 3.2 cell/mg, p = 0.03, **Figures 3G–I**).

### Neutrophil depletion reverses sepsis-induced muscle atrophy and weakness

**Figure 4:**
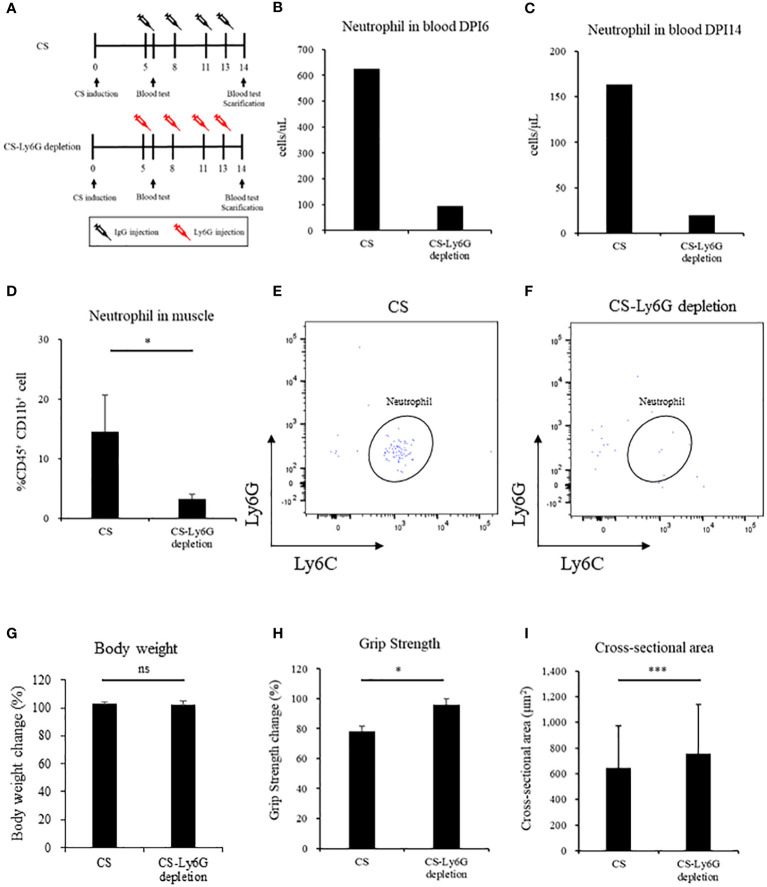
Neutrophil depletion reverses sepsis-induced muscle atrophy and weakness in mice. **(A)** Shema showing study design, **(B, C)** Number of neutrophil in blood in Day 6 and Day 14, (n = 1 in each group) **(D)** Percentage of neutrophils in muscle (n = 3–4 in each group), **(E, F)** FACS image 14 days after CS induction and CS-Ly6G depletion (n = 1 in each group) **(G)** Body weight 14 days after CS induction **(H)** Grip strength in 14 days after CS induction **(I)** Muscle cross-sectional area 14 days after CS induction. n = 5 in each group. Statistical significance versus CS at *P < 0.05, ***p < 0.001, ns = not significant (*p* > 0.05).

We depleted muscle-localized neutrophils by using Ly6G-specific monoclonal antibodies (**Figure 4A**). Neutrophils in the blood decreased in anti-Ly6G-treated CS mouse in one day after the CS induction (CS vs. CS-Ly6G depletion: 626.1 vs. 95.8 cells/uL, n = 1, 1) and at the time of scarifice (CS vs. CS-Ly6G depletion: 163.9 vs. 19.8 cells/uL, n = 1,1) (**Figures 4B, C**). In anti-Ly6G-treated CS mice, the percentage of neutrophils in the muscle was lower than in the CS mice (CS vs. CS-Ly6G depletion: 14.5% ± 6.1% vs. 6.1% ± 0.8%, p = 0.03, n = 3, 4, **Figure 4D**) with typical FACS image 14 days after induction (**Figures 4E, F**). Contrary to body weight (**Figure 4G**), grip strength was higher at Ly6G-depleted CS mice than CS mice (CS vs. CS-Ly6G depletion: 78.1% ± 3.8% vs. 96.2% ± 3.8%, p = 0.02, n = 5, 5, **Figure 4H**). Muscle atrophy was attenuated in Ly6G-depleted CS mice than CS mice (CS vs. CS-Ly6G depletion: 646.5 ± 327.8 μm^2^ vs. 755.6 ± 384.8 μm^2^, p < 0.001, **Figure 4I**). No mortality was observed till day 14 in five neutrophil-depleted mice. These results indicate that muscle-localized neutrophils contributed to muscle atrophy.

### Sepsis induces NETs in muscle

**Figure 5:**
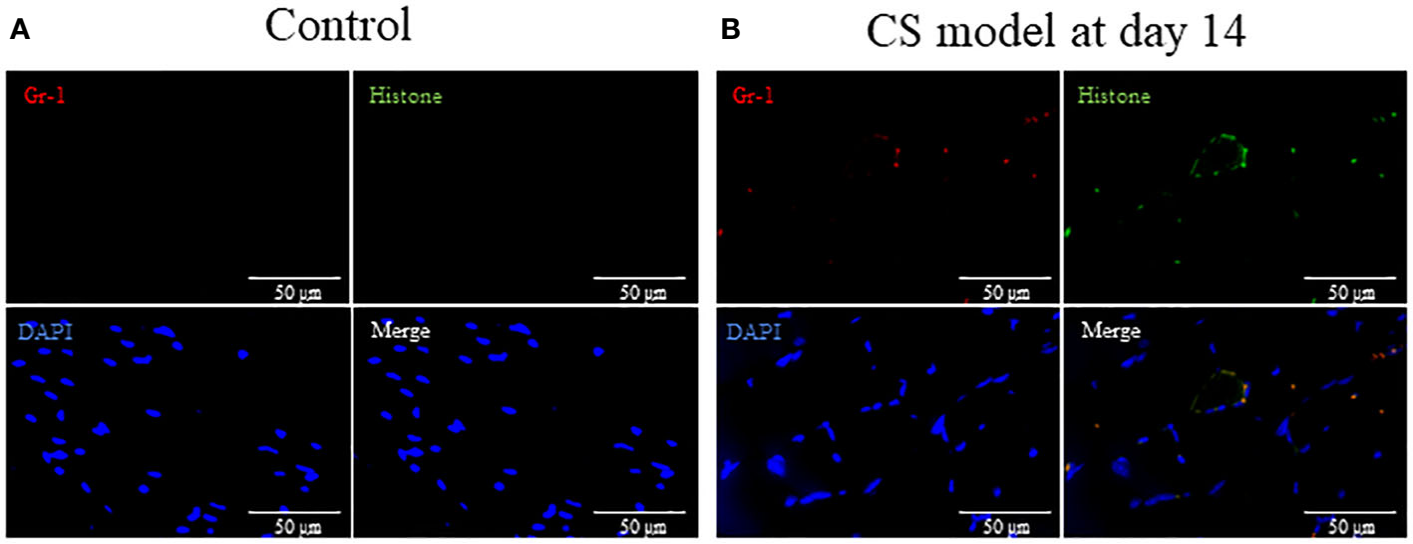
Sepsis induces NETs in muscle in mice. **(A)** In tibialis anterior muscle section, neutrophils, degranulation, and the nucleus are stained by Gr-1 (red), histone (green), and DAPI (blue), respectively. Gr-1, histone, and DAPI colocalization represent NETs. **(A)** No NETs were found in the control group. **(B)** At 14 days after CS injection, NETs were visually observed in the CS model. NETs: neutrophil extracellular traps, DAPI: 6-diamidino-2-phenylindole. n = 1 in each group.

Finally, we evaluated NETs formation in muscle to determine how neutrophils infiltrating the muscle affect the muscle. We compared the histologic changes in the control and CS-injected mice (Day14) to determine the differences in NETs formation. Gr-1, histone, and DAPI colocalization representing NETs was observed in the muscle in CS-injected mice (day 14), whereas those were not detected in the control (**Figure 5**).

## Discussion

In this study, we reveal that sepsis induces neutrophil infiltration in muscles in the subacute phase, leading to muscle atrophy and weakness in a CS-dose dependent manner. Furthermore, migrated neutrophils form NETs in muscles. We also demonstrated that neutrophil depletion reverses sepsis-induced muscle atrophy and weakness. These findings indicate that infiltrated neutrophils in muscles may play a critical role in sepsis-induced muscle weakness and atrophy.

Muscle-localized neutrophils might be associated with muscle atrophy. In a previous study, neutrophil levels were higher in the skeletal muscle of elderly mice, which was thought to be a cause of muscle atrophy (16). Likewise, previous studies have reported that neutrophil depletion decreased muscle atrophy in a non-sepsis model, such as hindlimb-unloading or cancer-induced muscle atrophy model (10, 17). However, such a relationship in sepsis has not been thoroughly investigated. Even though neutrophils are essential in a host’s defense against infection in sepsis, infiltration of neutrophils in the muscle is harmful, as it can induce the reduction of muscle volume and strength. This is the first time, to the best of our knowledge, it has been reported that muscle-localized neutrophils are likely contribute to muscle atrophy and weakness following sepsis.

Muscle-localized neutrophils are considered to release inflammatory cytokine, proteases, and free radicals, leading to exacerbated muscle atrophy (18, 19). Furthermore, we found NETs were prominent in muscle on neutrophil-peaked day 14, suggesting that NETs may be associated with muscle atrophy and weakness. NETs play an important role for infection control, but it also causes tissue injuries by increasing pro-inflammatory cytokines (20, 21).

Another new finding in this study is the difference of the timing in neutrophil elevation between blood and muscle tissue. Neutrophils are rapidly elevated in the blood in acute phase after sepsis, while those are elevated in muscle tissue in subacute phase after sepsis in this study. It is reasonable that rapid increase in neutrophil counts in the blood are equipped with antimicrobial agents to fight microbes on the front lines, however, it is still unclear why and how neutrophils migrated in muscle in the subacute phase of sepsis. Generally, neutrophil survival is usually limited to a few hours (22), therefore, we spectates that there is some mechanisms to extend neutrophil survival including suppression of their apoptosis. In previous study, apoptosis of neutrophils is suppressed in inflammatory tissue (23), and the neutrophils’ survival prolongs only for days in inflammation (24), it is unlikely that neutrophils remained for two to three weeks after sepsis was induced. Additionally, some neutrophil attractant cytokines might be released in muscle including chemokines, chemotactic lipids, formyl peptides, and complement anaphylatoxins (7). To address the mechanism of neutrophil migration to muscle, we examined expression of CXCR2 and CCR2 of neutrophils in muscle after sepsis, which are known as neutrophil migration factors. However, we could not find a significant increase of them in subacute phase after sepsis (Data not shown). Further study is needed to address the change and mechanism of neutrophil survival and the mechanism of neutrophil migration to muscles after sepsis.

There are several limitations to our study. First, neutrophil depletion and mortality were not thoroughly investigated. Although no mortality was observed till day 14 in five neutrophil-depleted mice, a previous study reported neutrophil dysfunction increases mortality (25). Second, we did not address the mechanism of neutrophil migration to muscle in sepsis. Third, we did not clarify the interaction of muscle atrophy and NETs. Fourth, we did not examine activation markers of neutrophils in muscle after sepsis. Fifth, we did not evaluate the relationship between neutrophils and other innate immune cells including monocytes and macrophages which also increased in muscle in the subacute phase. Further studies are needed to elucidate these points. Although our study contains several limitations, our finding could help to address the mechanism of this prolonged inflammatory state and muscle atrophy. In the future research to identify a chemoattractant that can orchestrate neutrophils in muscle will lead to a pharmacological intervention to eradicate muscle-specific immune cells.

## Conclusions

Sepsis causes infiltration of neutrophils in muscle and muscle atrophy and weakness. Depletion of neutrophils in muscle reverses sepsis-induced muscle atrophy and weakness. These results suggest that neutrophils may play a critical role in sepsis-induced muscle atrophy and weakness.

## Data availability statement

The raw data supporting the conclusions of this article will be made available by the authors, without undue reservation.

## Ethics statement

The animal study was reviewed and approved by Committee on the Ethics of Animal Experiments of Kobe University Graduate School of Medicine (P210704).

## Author contributions

NN and SI took part in study concept. NN, YM, and YO carried out the experiments. NN, KY, SI, and JK obtained research funding. YO, NM, KF, SI, and JK supervised the project. NN drafted the manuscript and all authors contributed to the revision. All authors contributed to the article and approved the submitted version.

## Funding

This study was partially supported by a crowdfunding project entitled the Muscle Atrophy Zero Project using the platform “Otsucle”. This study was partially supported by JSPS KAKENHI Grant Numbers JP20K17899.

## Acknowledgments

The authors thank people who sup-ported the nonprofit crowdfunding of Muscle Atrophy Zero Project, which aims to prevent muscle atrophy in critically ill patients.

## Conflict of interest

The authors declare that the research was conducted in the absence of any commercial or financial relationships that could be construed as a potential conflict of interest.

## Publisher’s note

All claims expressed in this article are solely those of the authors and do not necessarily represent those of their affiliated organizations, or those of the publisher, the editors and the reviewers. Any product that may be evaluated in this article, or claim that may be made by its manufacturer, is not guaranteed or endorsed by the publisher.
