# Supplementary material for: Sepsis causes neutrophil infiltration in muscle leading to muscle atrophy and weakness in mice
Source: Front Immunol. 2022 Oct 31;13:950646. doi: 10.3389/fimmu.2022.950646 (PMC9659852; doi:10.3389/fimmu.2022.950646)
Supplement: Supplementary file 1 [file DataSheet_1.docx]

Table S1. Antibody list for FACS analysis

| Antibody | Source | Clone | Catalog | Dilution |
| --- | --- | --- | --- | --- |
| Pacific Blue™ anti-mouse CD45 Antibody | BioLegend | S18009F | 157211 | 1/100 |
| Brilliant Violet 510™ anti-mouse/human CD11b Antibody | BioLegend | M1/70 | 101245 | 1/100 |
| PE/Cyanine7 anti-mouse F4/80 Antibody | BioLegend | BM8 | 123113 | 1/100 |
| PE anti-mouse Ly-6G/Ly-6C (Gr-1) Antibody | BioLegend | RB6-8C5 | 108407 | 1/100 |
| PerCP/Cyanine5.5 anti-mouse Ly-6G Antibody | BioLegend | 1A8 | 127615 | 1/100 |
| APC/Cy7 anti-mouse Ly6C Antibody | BioLegend | HK1.4 | 128015 | 1/100 |
| FITC anti-mouse CD192 (CCR2) Antibody | BioLegend | SA203G11 | 150607 | 1/100 |
| APC anti-mouse CD182 (CXCR2) Antibody | BioLegend | SA044G4 | 149311 | 1/100 |

Figure S1. Gating strategy for Fluorescence-Activated Cell Sorting


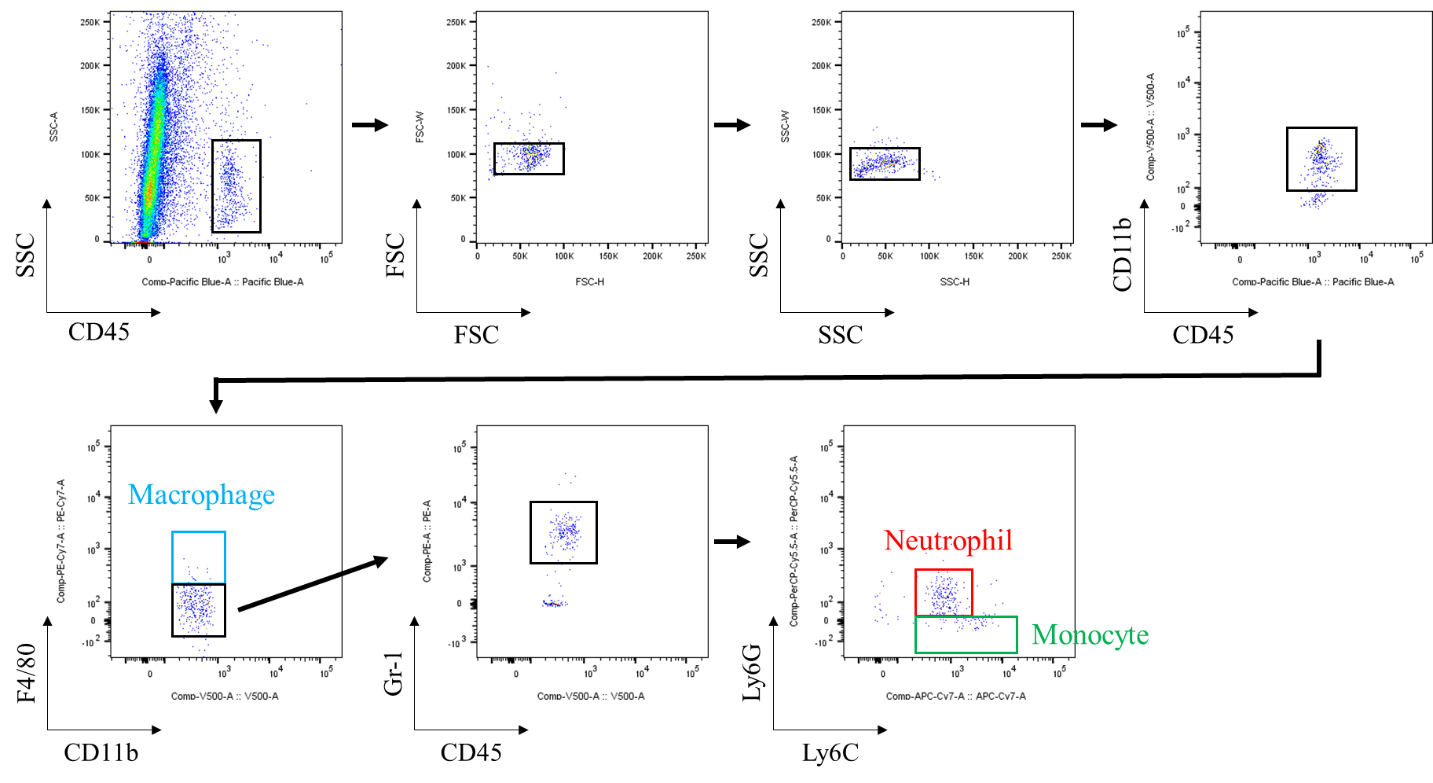


Figure S2. Survival rate


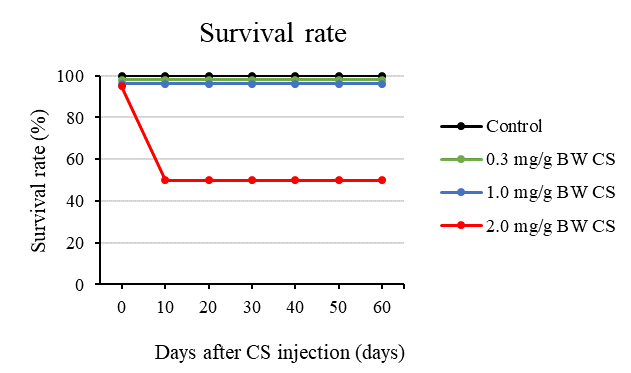


Figure S3. The longitudinal change of Gr-1^+^ cells in muscle following sepsis induction


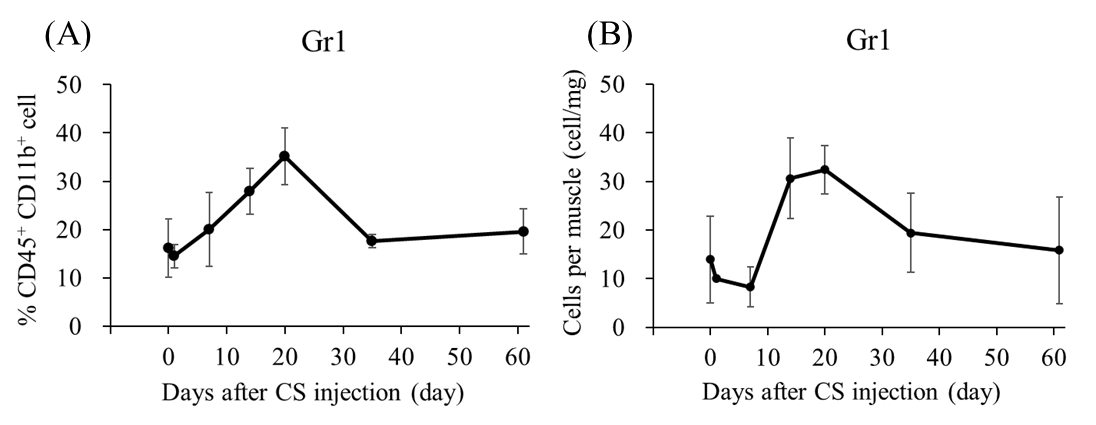


1. The percentage change of Gr-1^+^ cells in CD45^+^ CD11b^+^ cells. (B) The number of Gr-1^+^ identified per muscle.

Figure S4. Typical image of Fluorescence Activated Cell sorting of Gr-1^+^ cells.


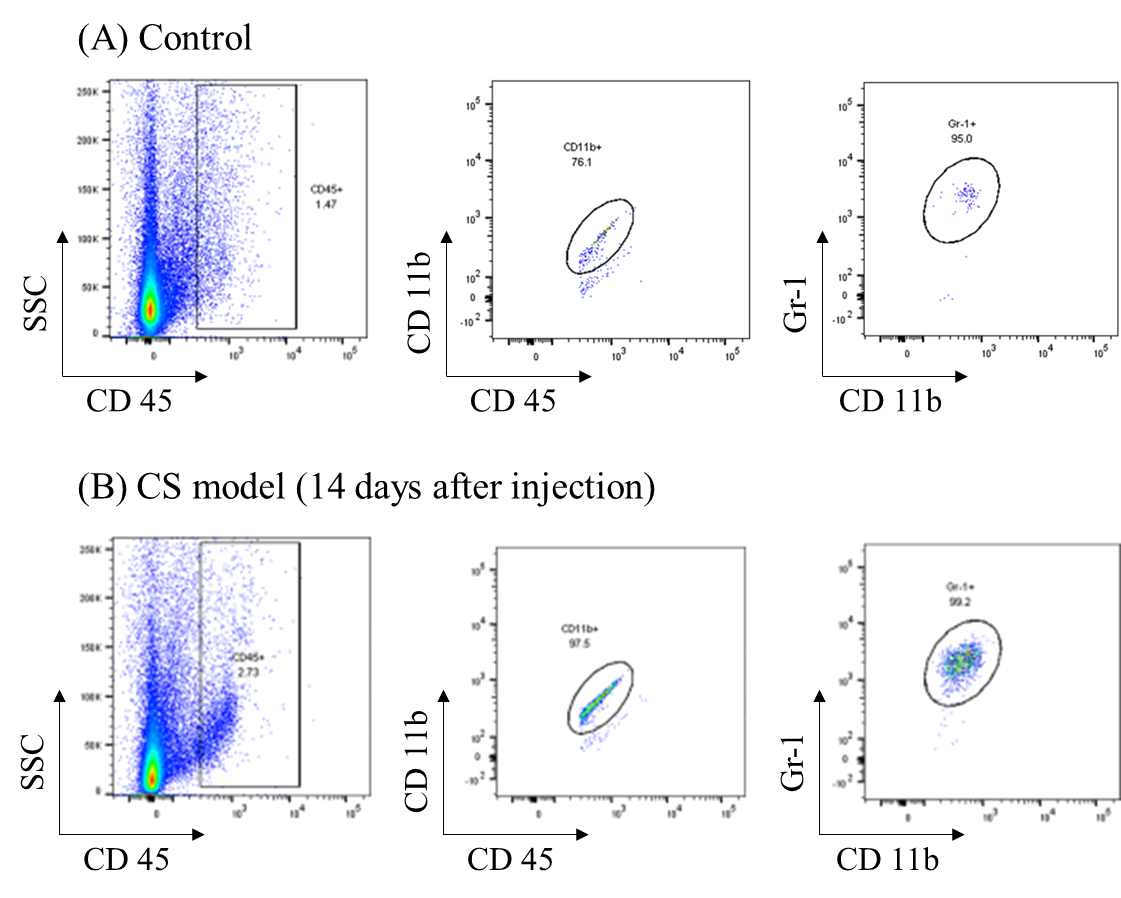


CD45^+^ CD11b^+^ Gr1^+^ myeloid cells were shown in (A) the control and (B) CS model 14 days after injection.

Figure S5. Immunostaining of Gr-1^+^ cells.


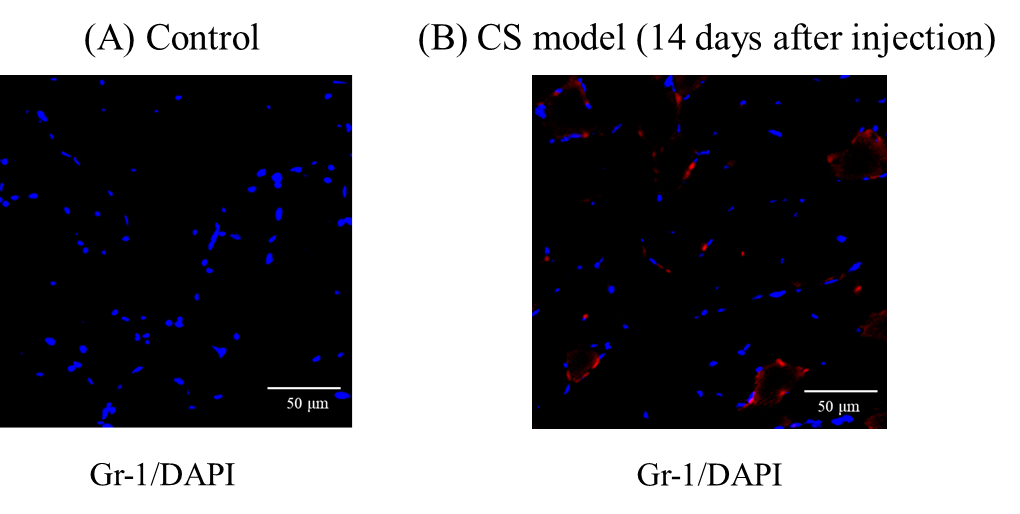


Immunohistochemistry revealed the presence of Gr-1^+^ cells in CS mice. Gr-1 (red), DAPI (blue)
